# Machine learning‐based classification of diffuse large B‐cell lymphoma patients by eight gene expression profiles

**DOI:** 10.1002/cam4.650

**Published:** 2016-02-11

**Authors:** Shuangtao Zhao, Xiaoli Dong, Wenzhi Shen, Zhen Ye, Rong Xiang

**Affiliations:** ^1^ School of MedicineCollaborative Innovation Center for BiotherapyNankai University94 Weijin RoadTianjin300071China; ^2^ Collaborative Innovation Center for BiotherapyNankai University94 Weijin RoadTianjin300071China; ^3^ Tianjin Key Laboratory of Tumor Microenvironment and Neurovascular RegulationTianjin300071China

**Keywords:** CHOP, diffuse large B‐cell lymphoma, gene expression profile, R‐CHOP, SVM

## Abstract

Gene expression profiling (GEP) had divided the diffuse large B‐cell lymphoma (DLBCL) into molecular subgroups: germinal center B‐cell like (GCB), activated B‐cell like (ABC), and unclassified (UC) subtype. However, this classification with prognostic significance was not applied into clinical practice since there were more than 1000 genes to detect and interpreting was difficult. To classify cancer samples validly, eight significant genes (*MYBL1, LMO2, BCL6, MME, IRF4, NFKBIZ, PDE4B*, and *SLA*) were selected in 414 patients treated with CHOP/R‐CHOP chemotherapy from Gene Expression Omnibus (GEO) data sets. Cutoffs for each gene were obtained using receiver–operating characteristic curves (ROC) new model based on the support vector machine (SVM) estimated the probability of membership into one of two subgroups: GCB and Non‐GCB (ABC and UC). Furtherly, multivariate analysis validated the model in another two cohorts including 855 cases in all. As a result, patients in the training and validated cohorts were stratified into two subgroups with 94.0%, 91.0%, and 94.4% concordance with GEP, respectively. Patients with Non‐GCB subtype had significantly poorer outcomes than that with GCB subtype, which agreed with the prognostic power of GEP classification. Moreover, the similar prognosis received in the low (0–2) and high (3–5) IPI scores group demonstrated that the new model was independent of IPI as well as GEP method. In conclusion, our new model could stratify DLBCL patients with CHOP/R‐CHOP regimen matching GEP subtypes effectively.

## Introduction

Diffuse large B‐cell lymphoma (DLBCL) is the most common non‐Hodgkin lymphoma including 30% of adult patients in western countries 1, but even higher percent in developing countries. It is heterogeneous in a wide spectrum of lymphoid neoplasms. The standard chemotherapy is CHOP (cyclophosphamide, doxorubicin, vincristine, and prednisone) or R‐CHOP (CHOP combined with rituximab), which produces a long‐term disease‐free survival of ~50% 2. Gene expression profiling (GEP) has been extensively used in the classification of DLBCL as an alternative microarray technology. It is the most well‐established method to distinct DLBCL subtypes with significant prognostic power 3, such as germinal center B‐cell like (GCB), activated B‐cell like (ABC), and unclassified (UC) subtype 4. ABC subtype patients with distinctive genes from activated B cells and plasma cells have a poor clinical outcome (5‐year survival rate, 30%), whereas GCB subtype patients express a signature of normal germinal center B cells with a more favorable overall survival (5‐year survival rate, 59%) 5. The amplifications of the *REL* loci, *BCL2* translocations, and hypermutations of the immunoglobulins loci are the typical characteristics of GCB subtype. However, a distinctive feature of ABC subtype is the constitutive activation of the nuclear factor *kB* pathway 6. With little loss of specificity or sensitivity, GEP can be defined by more than 1000 distinct genes capable of accurately subtyping DLBCL 7, which undoubtedly led to continued investment in personalized medicine opportunities in DLBCL 8.

However, GEP technology for routine clinical practice is challenging for expensive and technical constraints, and the need for intensive bioinformative analysis. In order to translate it as a manageable set, several methods have been reported in recent years based on immunehistochemical stains tissue microarray technique. Hans et al. proposed the primary algorithm based on the three‐protein markers: neprilysin or common acute lymphocytic leukemia antigen (CD10), B‐cell lymphoma 6 (BCL6), and multiple myeloma oncogene 1 (MUM1), which could divide patients into two groups (GCB and Non‐GCB) with distinct prognosis. But, this method had a low concordance with GEP analysis (GCB, 71%; and Non‐GCB, 88%) for patients with CHOP regimen and inconsistent results with patients treated by R‐CHOP in the prognostic relevance 9. Another algorithm reported by Choi et al. also had a low concordance (83%) with GEP analysis for discrimination between GCB and Non‐GCB subtypes by integrating another two new markers: forkhead box protein P1 (FOXP1) and serpin A9/germinal center expressed transcript 1 (GCET1) 10. C Visco et al. developed an effective method called Visco‐Young algorithm, which had high concordance (92.6%) between patients with GCB and ABC gene profiles 9. And this algorithm that was composed of MME, FOXP1, and BCL6, exhibited strong independent prognostic power in DLBCL patients treated with R‐CHOP. Although it was becoming more and more utilized in clinical work, some existing defects impacted on the development of this method. There were many steps that affect the dyeing result in the process of immunehistochemical staining. It was strongly influenced by the experimenter technology level, especially in the results to determine stronger subjectivity.

Today, new high‐throughput technologies have allowed a better understanding of the molecular basis of this disease. We used machining learning method to screen and obtain eight specific markers, including *MYBL1, LMO2, BCL6, MME, IRF4, NFKBIZ, PDE4B*, and *SLA,* to stratify DLBCL patients through the significantly different expression among GCB, ABC, and unclassified types. Finally, we developed an effective model match with high concordance (94%) with GEP analysis. The new model demonstrated strong independent prognostic power, which was most equivalent to that of GEP analysis in a large cohort of DLBCL patients treated with CHOP/R‐CHOP chemotherapy.

## Methods

### Training data and validation data

The raw files were downloaded from GEO database with the same platforms GPL570 (Affymetrix Human Genome U133 Plus 2.0 Array, Santa Clara, CA, USA) and the expression of genes were normalized by the average of three house‐keeping genes (ACTB, GAPDH, and LDHA). A group of 414 patients from GSE10846 were treated as training set and another 855 patients from GSE19426, GSE53786, GSE56315, and GSE31312 were as two validated sets. All the DLBCL cases had been published between September 2010 and April 2015, which were selected on the basis of the available GEP results and clinical data. All diagnoses were confirmed on the basis of WHO classification criteria. In order to test the efficacy in predicting survival in another independent series of cases, a part of patients (*n *=* *119, GSE53786; *n *=* *475, GSE31312) from validated set were applied into the new model with the same selection criteria as those for the first cohort GSE10846. Of these three data sets (GSE10846, GSE53786, and GSE31312), 225 patients had been treated with CHOP and 778 with R‐CHOP. Clinical characteristics at presentation for the validated set were similar to the training data set in terms of age (≥60 in 54%, *P *=* *0.012), stage III—IV (53%, *P *=* *0.651) or IPI (0–2 in 50%, *P *=* *0.830), except for gender. We could not obtain the gender from the validation set.

### Cutoff establishment

We avoided cutoff values based on the mean or median expression because our gene expression had a non‐Gaussian distribution. Instead, we identified the point on the curve corresponding to the maximum sensitivity and specificity for each gene to classify a DLBCL as either of GCB or ABC type according to GEP analysis by calculating the Youden index from our ROC curves.

### Receiver–operating characteristic (ROC) curve analysis to assess discriminatory accuracy of each gene

The ROC curves allowed us to visualize the sensitivity and specificity of the eight genes (*MYBL1, LMO2, BCL6, MME, IRF4, PDE4B, NFKBIZ*,* and SLA*) in assigning patients to GCB or Non‐GCB subtype before further categorization. The performance of each gene could be quantified by the area under the ROC curve. All patients were classified separately as GCB or Non‐GCB based on the cutoff scores from both data sets and the eight genes.

### Statistical analysis

We obtained 11 significant genes from 57 genes published on reviews among GCB, ABC, and unclassified subtype in 414 patients of GSE10846 with Kruskal–Wallis H test and Nemenyi test. Meanwhile, we had 20 out of 57 genes to discriminant GCB, ABC, or unclassified subtype with forward stepwise discriminant analysis. As a result, eight common genes (*MYBL1, LMO2, BCL6, MME, IRF4, PDE4B, NFKBIZ*,* and SLA*) were produced from the two methods, which evaluated the correlation with GCB or Non‐GCB subtype by Correspondence Analysis. We then selected the best method for constructing the model from seven Machine Learning methods (Decision tree, Random forest, support vector machine, Fisher discriminant analysis, Nearest Neighbor, Bagging, and Adaboost). We intended to choose the method with the minimum error rate. At last, we develop the SVM model with the R package “e1071” including eight gene markers. The actuarial probability of overall survival (OS) was determined using the Kaplan–Meier method, and differences were compared using the log‐rank test. A Cox proportional hazards model was used for multivariate analysis. All variables with *P* < 0.05 were considered to be statistically significant.

## Results

### The significant genes and the optimum method were screened in microarrays data

**Figure 1:**
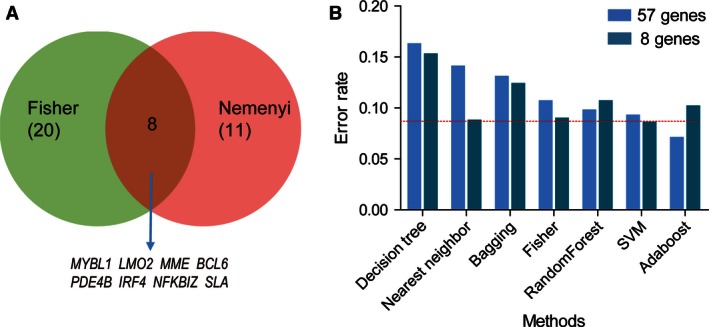
Eight significant genes and support vector machine (SVM) method were selected to construct a new model. (A) Overlaps between genes selected by Fisher discriminant and that by Nemenyi statistical analysis among GCB‐, ABC‐ and unclassified diffuse large B‐cell lymphoma (DLBCL) patients. Venn diagram showed the overlap genes which could stratify the DLBCL patients into GCB and Non‐GCB subtypes. (B) Histogram of the classified error rate of seven machine learning methods between the expression of 57 genes and eight genes with fivefold cross validation in 414 DLBCL patients. The error rate of SVM was the minimum within eight genes model, also smaller than 57 genes model.

To select the most befitting factors for developing a classification model, we obtained 57 genes correlated with prognosis or drug resistance of DLBCL from previous efforts of others (Table S1.), which were measured with microarrays in GSE10846. We found 11 significant genes among the subtypes (GCB, ABC, and Unclassified type) of DLBCL with the different expression analysis. Meanwhile, a Fisher discriminant analysis was performed with GEP subtypes as a dependent variable, which included age, gender, Ann arbor stage, genotype, and the expression of 57 genes except for IPI (since IPI was overlapped with age and Ann arbor stage). As a result, 20 genes were left for dividing the three subtypes of DLBCL. Finally, we got eight common genes from Fisher and different expression analysis (Fig. 1A).

In order to obtain the best method for constructing the model, we compared the discriminant error rate between the expression of 57 genes and eight genes with fivefold cross validation in seven Machine Learning methods (Fig. 1B). At last, we chose the support vector machine (SVM) method to construct a classification model for the minimum error rate.

### Distribution and prognostic significance of the expression of each gene marker

**Figure 2:**
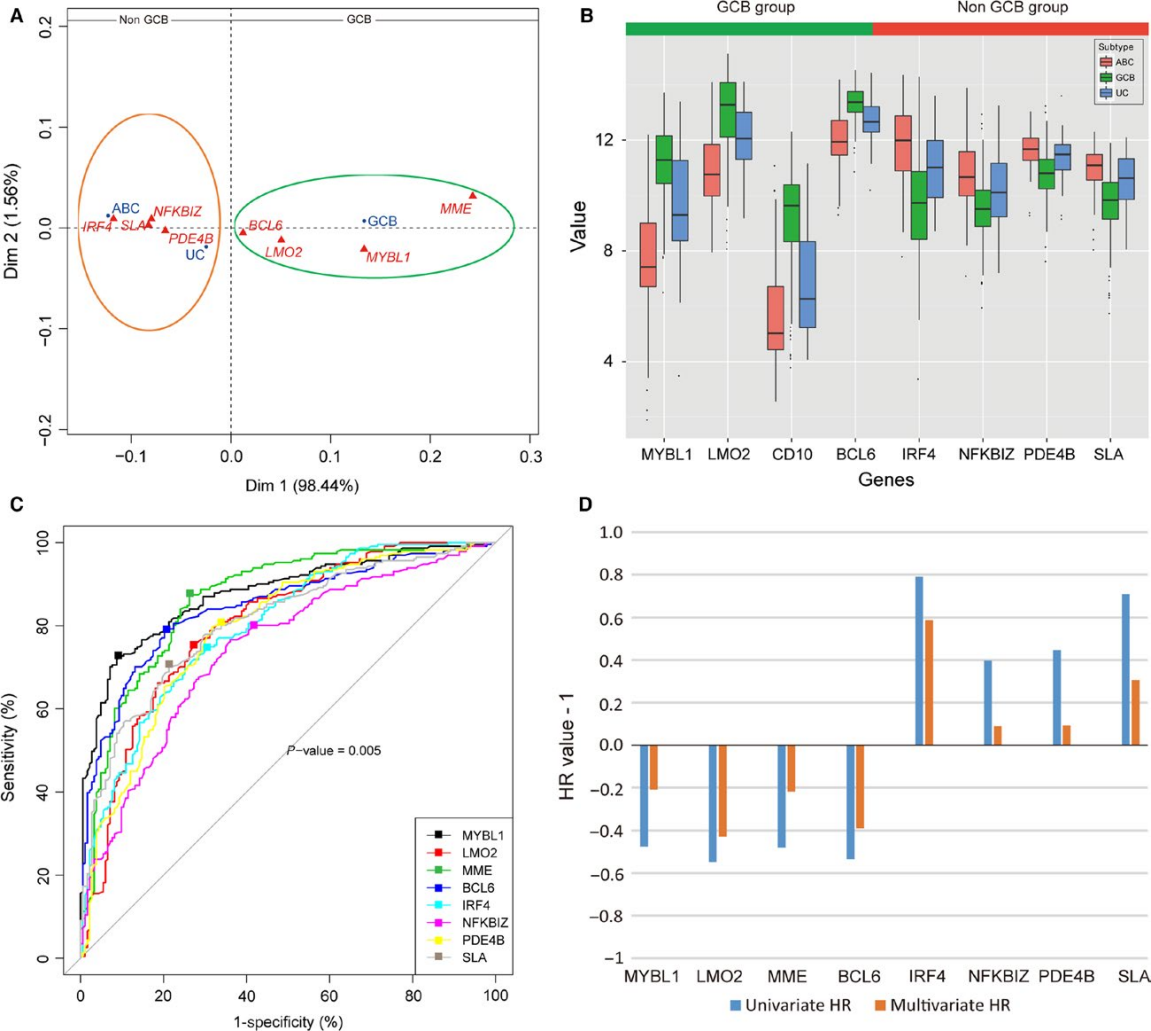
Explore the eight significant genes' distribution with Correspondence Analysis (CA) and cutoff values with receiver–operating characteristic (ROC) analysis in GSE10846. (A) Plot of first and second axes (Dim 1*2) for the classification of diffuse large B‐cell lymphoma (DLBCL) subtypes and eight significant genes according to their variables. Projection of the variables in principal plane 1*2: the first and the second dimensions present, respectively, 98.44 and 1.56% of the total inertia. The sharps of the clouds suggest a repulsion between two groups (GCB and Non‐GCB) of variables. Group1: *MYBL1, LMO2, BCL6*, and *MME* seem to characterize GCB subtype patients in the factorial design (green circle). Group2: On the opposite side, the Non‐GCB patients were characterized by the presence of *IRF4, PDE4B, NFKBIZ*, and *SLA* (dark orange circle). (B) Different expression of eight significant genes were divided into two groups. Eight genes' expression levels in GCB, ABC, and unclassified (UC) subtype. Expression levels are presented as boxplots and were compared using the Mann–Whitney test and the Nemenyi test (all *P*‐values < 0.05). (C) Calculate the Youden index from the ROC curves. The points stand for cutoff values were identified corresponding to the maximum sensitivity and specificity to classify a DLBCL as GCB or Non‐GCB type according to Gene expression profiling (GEP) analysis. (D) Compare the hazard ratio (HR) of the eight genes from univariate analysis and multivariate analysis by using Cox regression. Both univariate and multivariate HR of four genes (*MYBL1, LMO2, MME*, and *BCL6*) were negative, and the left four genes (*IRF4, NFKBIZ, PDE4B* and *SLA*) were with positive value.

To explore the correlation between the eight common genes and the three subtypes of DLBCL, we conducted Correspondence Analysis with the expression of eight genes in GSE10846. We found that four genes (*MYBL1, LMO2, BCL6*, and *MME*) around GCB type, and another four genes were adjacent to the ABC and Unclassified type, which were also named Non‐GCB subtype (Fig. 2A). Then, we observed the expression levels of the eight selected genes and we found that the four genes included in GCB group had a significant higher expression in GCB‐DLBCL cases as compared to the other genes. Oppositely, the ABC‐DLBCL patients were characteristic as significant higher expression of the four genes in Non‐GCB group. Equally, the eight genes had the middle status expression in unclassified subtype (Fig. 2B). All the analysis showed that it was impossible for predicting the subtypes of DLBCL with the expression of eight common genes.

The cutoffs determined with the Youden index were not intended to predict survival, but only to determine patients as having either GCB or Non‐GCB subtype. With the Youden index, we established the positivity cutoffs for *MYBL1* (value = 9.3; specificity 90.7%; sensitivity, 72.7%), *IRF4* (value = 10.7; specificity 69.9%; sensitivity, 74.9%), *LMO2* (value = 12.3; specificity 72.1%; sensitivity, 75.8%), *BCL6* (value = 13; specificity 79.2%; sensitivity, 79.2%), *NFKBIZ* (value = 10.08; specificity, 63.9%; sensitivity, 76.6%), *SLA* (value = 10.5; specificity 78.7%; sensitivity, 70.1%), *MME* (value = 8.5; specificity 73.8%; sensitivity, 87.4%), and *PDE4B* (value = 11.5; specificity 68.3%; sensitivity, 79.7%)(Fig. 2C). Expression above these cutoffs for *MYBL1* was observed in 232 (56%) patients, *LMO2* in 213 (51%), *MME* in 166 (40%), *BCL6* in 183 (44%), *IRF4* in 226 (55%), *NFKBIZ* in 202 (49%), *PDE4B* in 155 (37%), and *SLA* in 204 (49%) (Table 1). As a result, we divided the expression of each marker into two subgroups (high and low group) in 414 patients according to the cutoff values (Table 1).

**Table 1:** Univariate and multivariate Cox regression analysis of 8 genes in GSE10846

Genes	Group	Cutoff value	Overall		Univariate*P* ‐value	Multivariate*P* ‐value	GCB		Non‐GCB		*P* ‐value
			*N*	%			*N*	%	*N*	%	
*MYBL1*											
	High group	≥9.3	232	56	0.0001	0.492	167	91.3	65	28.1	0.000
	Low group	<9.3	182	44			16	8.7	166	71.9	
*LMO2*											
	High group	≥12.3	213	51	2.5E–07	0.009	141	77.0	72	31.2	0.000
	Low group	<12.3	201	49			42	23.0	159	68.8	
*MME*											
	High group	≥8.5	166	40	4.15E–05	0.327	135	73.8	31	13.4	0.000
	Low group	<8.5	248	60			48	26.2	200	86.6	
*BCL6*											
	High group	≥13	183	44	1.50E–06	0.038	138	75.4	45	19.5	0.000
	Low group	<13	231	56			45	24.6	186	80.5	
*IRF4*											
	High group	≥10.7	226	55	0.000531	0.023	55	30.1	171	74.0	0.000
	Low group	<10.7	188	45			128	69.9	60	26.0	
*NFKBIZ*											
	High group	≥10.08	202	49	0.0351	0.801	38	20.8	164	71.0	0.000
	Low group	<10.08	212	51			145	79.2	67	29.0	
*PDE4B*											
	High group	≥11.5	155	37	0.0381	0.721	19	10.4	136	58.9	0.000
	Low group	<11.5	259	63			164	89.6	95	41.1	
*SLA*											
	High group	≥10.5	204	49	0.00129	0.220	41	22.4	163	70.6	0.000
	Low group	<10.5	210	51			142	77.6	68	29.4	

Next, we concerned the prognostic significance in univariate and multivariate analysis of the eight genes in the two subgroups. And we discovered that 414 patients from the data set were with significant prognosis in univariate analysis of OS (*P* < 0.05). What is more, the expression of the eight genes were much significantly different (*P *=* *0.000) between GCB and Non‐GCB subtype from GEP analysis in high and low group. In the high group of four genes (*MYBL1, LMO2, MME*, and *BCL6*), the number of patients with GCB subtype were significantly more than Non‐GCB subtype (Table 1), which indicated that patients with high expression value of these genes would be favorable prognosis. On the contrary, the number of Non‐GCB subtype patients were significantly more in the high group of the left four genes (*IRF4, NFKBIZ, PDE4B,* and *SLA*), which demonstrated these patients would be poor clinical outcome(Table 1). Then, these results were validated in Figure S1, *MYBL1, LMO2, MME*, and *BCL6* of expression above the cutoffs were significantly associated with preferable Overall Survival (OS) result, however, the expression of the other four genes above the cutoffs were instead significantly associated with poorer OS (*P* < 0.05). However, we only obtained three significant genes *LMO2* (*P *=* *0.009), *BCL6* (*P *=* *0.038), and *IRF4* (*P *=* *0.023) from the multivariate analysis of OS by using Cox regression (Table 1). Meanwhile, the hazard ratios (HR) of the eight markers from univariate analysis and multivariate Cox regression analysis were in accordance with above (Fig. 2D). The positive HRs (*IRF4, PDE4B, NFKBIZ*,* and SLA*) were associated with the relative expression value of gene which indicated poor prognosis, and the negative HRs (*MYBL1, LMO2, MME* and *BCL6*) were associated with the relative value of gene, which correlated with good prognosis. Generally, all the prognostic analysis indicated that the eight genes were certainly used to construct the SVM mode to stratify subtypes of DLBCL.

### Molecular classification model was developed to stratify DLBCL patients

**Figure 3:**
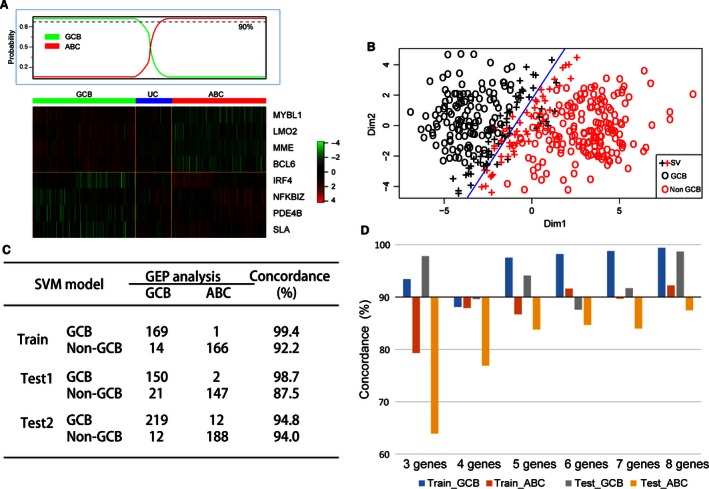
Develop the new classified model with high concordance with Gene expression profiling (GEP) analysis. (A) Heat map of hierarchical clustering of GEP in 414 patients with diffuse large B‐cell lymphoma (DLBCL). Patients as GCB‐DLBCL on the left show all the patients express selected genes. Similarly, patients stratified as ABC‐DLBCL on the right express hierarchically selected genes. Patients in the middle area could not be stratified by GEP analysis and considered as unclassifiable subtype (UC). (B) Distribution tails of eight markers define samples in the GCB and Non‐GCB subtypes with support vector machine (SVM) model. The classification threshold as shown (blue line) in the plot were defined using SVM algorithm. And the error rate of discrimination was 5.6% in total. SV: support vector. (C) Concordance between GEP analysis and eight genes model in 350 training cases, 320 validated cases of Test1 and 431 validated cases of Test2 who were classified by GEP either as GCB or ABC (excluding unclassified cases *n *=* *64 in training cohort, *n *=* *60 in validated Test1 and *n *=* *44 in validated Test2). (D)Compare concordances between GEP analysis and different genes models in training and testing data set. The model of three genes was composed of *LMO2, BCL6*, and *IRF4*. And the model of four genes was integrating another gene *SLA* on base of three genes model. Similarly, the models of 5–8 genes were added *PDE4B, NFKBIZ, MME*, and *MYBL1* into four genes model one by one in sequence according to the hazard ratio value.

We procured *n *=* *414 DLBCL patients with chemotherapy of CHOP or R‐CHOP from GSE10846, which were classified into GCB (183, 44.2%), ABC (167, 40.6%), or unclassifiable (64, 15.2%) cases by GEP analysis (Fig. 3A). We conducted the classified analysis with the gene expression of eight significant genes in SVM model. As result, the eight‐marker model correctly characterizes 94.4% of patients as either GCB or Non‐GCB subtype according to GEP analysis in training datasets of GSE10846 (Fig. 3B). According to the eight‐marker model, 170 patients (41.1%) had a GCB phenotype and 244 (58.6%) had a Non‐GCB phenotype. The 64 unclassifiable cases were assigned to the GCB (9) or the Non‐GCB (55) subgroups by the new model. Furthermore, our new model had a concordance with GEP results of 99.4% or 99.2% for the 350 patients classified by GEP as having either GCB (1 mismatch out of 170 patients) or ABC (14 mismatches out of 180 patients) disease (Fig. 3C).

It would be greater to have fewer genes as possible to be analyzed to make the new model more competitive. In order to validate the eight genes group as the “less‐gene‐possible” combination, we constructed the SVM model with the three significant genes (*LMO2, BCL6*, and *IRF4*) from multivariate analysis (Table 1). As a result, the three‐gene model had a high concordance for GCB subtype (Train_GCB, 93.4%; Test_ABC, 97.8%), but a low concordance for ABC subtype (Train_GCB, 79.3%; Test_ABC, 63.9%) with GEP analysis for patients in training data sets and testing data sets **(**Fig. 3D**)**. Then, we integrated a gene into the model according to the multivariate hazard ratio value (high to low). Finally, we discovered that the eight genes had more concordance with GEP analysis than the other five models between GCB and Non‐GCB subtype in training and testing data sets, respectively **(**Fig. 3D**)**. Generally, we concluded that the eight‐gene SVM model was the “less‐gene‐possible” combination to divide the subtypes of DLBCL effectively**.**

**Figure 4:**
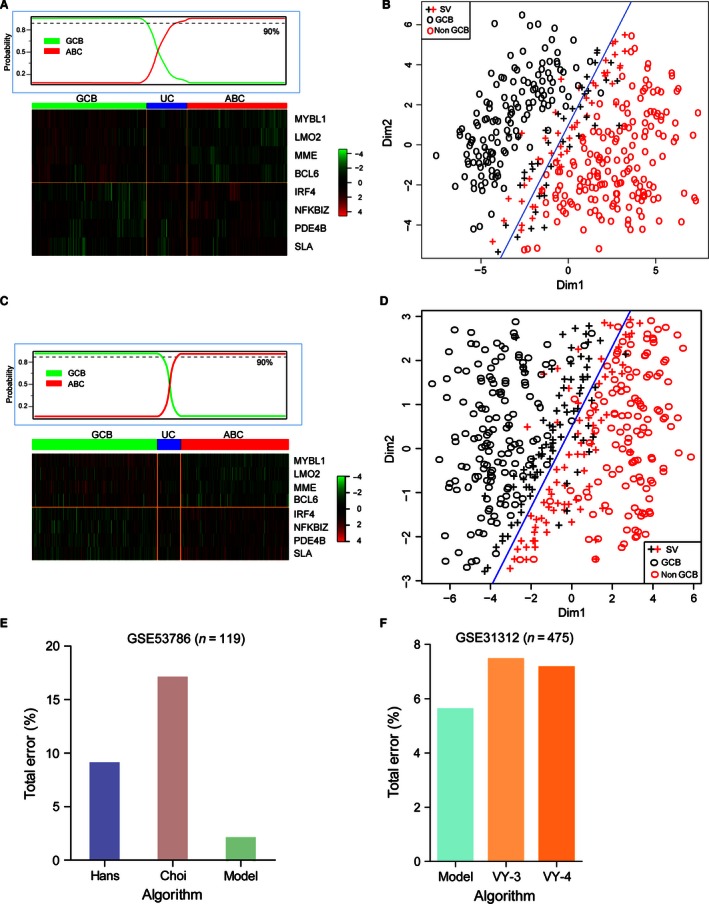
Validation of diffuse large B‐cell lymphoma (DLBCL) subgroups using the new model in 380 patients. Heat map of hierarchical clustering of Gene expression profiling (GEP) in validated cohort1 (A) and cohort2 (C) with DLBCL. Cases as GCB, ABC or unclassified (UC) subtype show the similarly relative expression with the training data within eight selected genes. Test the efficacy of the new model in stratifying of 380 patients in cohort1 with discriminant error rate 9% (B) and 475 patients in cohort2 with discriminant error rate 5.6% (D) as GCB or Non‐GCB subtype. Compare the classified error rate of the new model with two IHC methods (Hans and Choi. Algorithm) in GSE53786 (E) and 3 or 4‐markers Visco‐Young algorithm in GSE31312 (F), the error rates of the SVM model were 2% and 5.6% in GSE53786 and GSE31312, respectively.

We confirmed the presence of the proposed new algorithm in previously published DLBCL cohorts including *n *=* *380 patients (GSE19426, GSE53786, and GSE56315) and 475 patients (GSE31312). We applied the new model to gene expression data from the validated patients and observed that the two validated cohorts could be divided into two subtypes, respectively, which had high concordance with GEP analysis as seen in GSE10846 (Figs. 3C and  4A–D). And the validated result suggested that our new model could be reproduced in other DLBCL cohorts effectively. In terms of error rates for the classification, our new algorithm compared favorably both with the Choi and Hans algorithms. The error rates were 2% for our new algorithm versus 9% for Hans algorithm and 14% for Choi algorithm in GSE53786 (Fig. 4E). Similarly, the error rate was lower in our SVM model (5.6%) than 3‐ or 4‐markers Visco‐Young algorithm (7.45% and 7.15%, respectively)^9^ in GSE31312 (Fig. 4F). All the results indicated that the SVM model based on eight genes profiles could be more feasible for clinical use as its higher accuracy in classification of DLBCL patients.

### The classification model was associated with clinical profiling

Clinical characteristics at presentation for the 414 CHOP or R‐CHOP‐treated patients with de novo DLBCL were stratified according to our proposed eight‐marker algorithm as shown in Table 2. Clinical variables were well balanced between GCB and Non‐GCB subgroups except for Performance Status, clinical stage and IPI risk scores. Patients with the Non‐GCB phenotype were significantly older (median age, 64.5 vs. 60 years), more advanced (57.8% vs. 48.3%, III—IVstage) and had higher IPI scores (33.7% vs. 22.9%; IPI 3–5) than patients with the GCB phenotype, as shown in Table 2.

**Table 2:** Clinical characteristics and their impact on survival of 414 diffuse large B‐cell lymphoma (DLBCL) patients treated with CHOP/R‐CHOP, then stratified by the SVM model as GCB or Non‐GCB group

	Overall		Univariate *P* ‐value	Multivariate *P* ‐value	GCB		Non‐GCB		*P* ‐value
	*N*	%			*N*	%	*N*	%	
Patients	414	100							
Age									
≥60	235	56.8	0.000	0.004	92	50.3	143	61.9	0.018
<60	179	43.2			91	49.7	88	38.1	
Gender									
Female	172	43.4	0.954	0.929	73	43.7	99	43.2	0.924
Male	224	56.6			94	56.3	130	56.8	
Stage									
I—II	188	46.3	0.000	0.027	90	51.7	98	42.2	0.047
III—IV	218	53.7			84	48.3	134	57.8	
LDH									
Normal	173	49.3	0.000	<0.0001	85	49.1	70	39.3	0.064
High	178	50.7			88	50.9	108	60.7	
Performance status									
0–1	295	75.8	0.000	0.016	142	84	153	69.5	0.001
2 or more	94	24.2			27	16	67	30.5	
*N* extra nodal sites									
0–1	353	92.2	0.028	0.461	153	93.9	200	90.9	0.287
2 or more	30	7.8			10	6.1	20	9.1	
IPI risk group									
0–2	228	71	0.000	0.013	108	77.1	120	66.3	0.034
3–5	93	29			32	22.9	61	33.7	

As shown in Table 2, univariate analysis for OS revealed that a high IPI score, age, stage, LDH ratio, extra nodal sites, and performance status were significantly associated with a shorter OS. In multivariate analysis using the Cox regression model, an IPI score 3–5 (hazard ratio, 2.437; 95% CI, 0.936–5.882; *P *=* *0.007), advanced stageIII—IV (hazard ratio, 1.159; 95% CI, 0.889–1.513; *P *=* *0.027), high LDH ratio (hazard ratio, 1.135; 95% CI, 1.067–1.208; *P *=* *0.000), high ECOG performance status (hazard ratio, 1.336; 95% CI, 1.055–1.694; *P *=* *0.016), and elder age (hazard ratio, 1.026; 95% CI, 1.008–1.043; *P *=* *0.004) were independent adverse prognostic factors for OS. Moreover, univariate and multivariate analysis were performed in the 119 patients treated with CHOP or RCHOP of the validation set GSE53786 and 475 patients with R‐CHOP treatment from GSE31312. Similar to the former, both older aged (GSE53786: HR, 4.367; 95% CI, 1.382–13.795; *P *=* *0.012; and GSE31312: HR, 1.537; 95% CI, 1.130–2.090; *P *=* *0.006) and high LDH ratio (GSE53786: HR, 6.005; 95% CI, 1.926–18.724; *P *=* *0.002, and GSE31312: HR, 1.172; 95% CI, 0.834–1.648; *P *=* *0.361) resulted in independent adverse prognostic factors for OS.

Gender might be correlated with prognosis of patients with DLBCL. Mustafa Yildirim et al. suggested that male gender to be a critical factor for a poor prognosis in DLBCL patients with rituximab‐containing regimens by analyzing 5635 patients from 20 studies with a meta‐analysis 11. And Carsten Muller et al. demonstrated that the elderly males benefited less from the R‐CHOP regimen than female was a gender‐dependent effect contributed to clearing rituximab faster by investigating the serum rituximab levels of 20 DLBCL patients 12. Meanwhile, our study validated above result by analyzing 414 DLBCL patients with univariate analysis and multivariate analysis, which demonstrated that gender was associated with poor prognosis (HR = 1.01) of DLBCL patients treated with CHOP/R‐CHOP, although they did not reach the significant level (*P *=* *0.954 and *P *=* *0.929, respectively). Generally, although gender had not been inclusive in the prognostic system of DLBCL patients, the elderly male patients should also receive more attention on the clinical treatment.

### Predictive power of the new model was similar with GEP analysis

**Figure 5:**
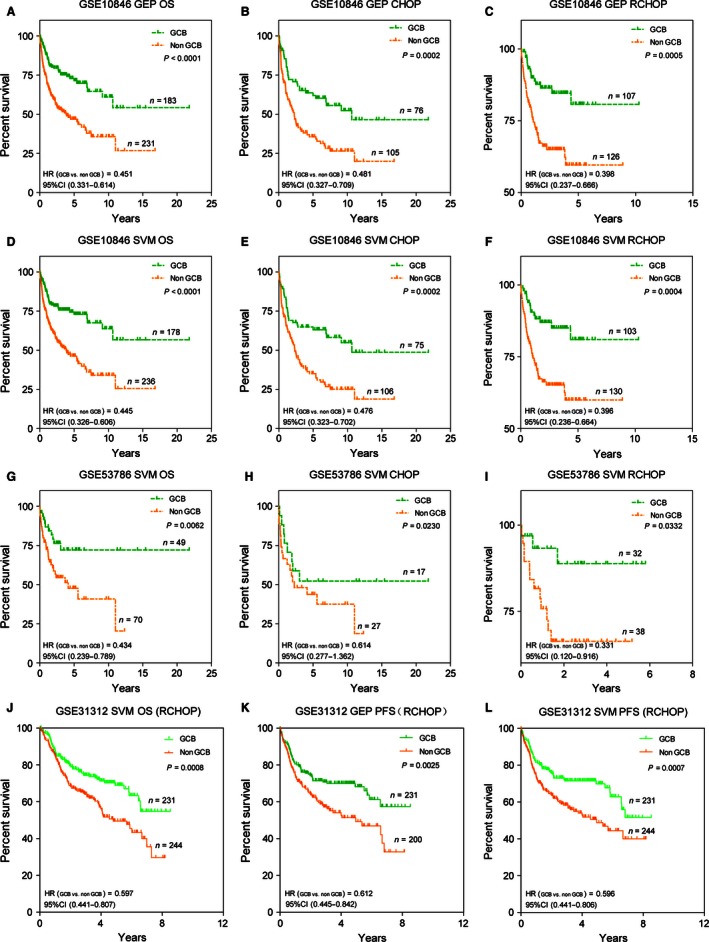
Overall survival (OS) analysis of CHOP/R‐CHOP‐treated DLBCL patients when stratified by Gene expression profiling (GEP) and the support vector machine (SVM) model. OS curve for 414 patients stratified by GEP results (A) and SVM model (D), and validated in 119 patients from GSE53786 stratified by SVM model (G). And OS curve for 181 patients treated with CHOP regimen and 233 patients with R‐CHOP stratified by GEP analysis (B, C) and SVM model (E, F), and validation set of OS in 119 patients treated with CHOP or R‐CHOP and another 475 patients treated with R‐CHOP stratified according to the SVM model (H‐J). PFS curves of 431 patients were classified by GEP method (K), 44 out of 475 unclassifiable patients were exclusive. And PFS curves of 475 patients stratified into GCB and Non‐GCB by the SVM model (L).

Median follow‐up was 2.38 years (range, 0–21.78 years). Overall, the five‐year OS was 57.71%. We found that there were no different outcomes in patients divided by GEP or the new model. As shown in Figure 5A, the five‐year OS was significantly different when patients were stratified by GEP method (71.48% for GCB vs. 46.87% for Non‐GCB; HR, 0.451; 95% CI, 0.331–0.614; *P *<* *0.0001). Analogously, the five‐year OS was significantly different when DLBCL patients were stratified by the eight‐marker model (72.94% for GCB vs. 46.29% for Non‐GCB; HR, 0.445; 95% CI, 0.326–0.606; *P *<* *0.0001; Fig. 5D). As there were 64 unclassified cases according to GEP subgroups and they could not be excluded in the clinical work, the use of the eight‐maker model allowed us to stratify the cases into two groups with different OS rates. However, the rates were not significantly different because of the small number of cases assigned to GCB subtype.

In order to confirm the reliability of our model in predicting survival, we verified it in the set of 119 patients from GSE53786 (HR, 0.434; 95% CI, 0.239–0.789; *P *=* *0.006; Fig. 5G) and 475 patients from GSE31312 (HR, 0.597; 95% CI, 0.441–0.807; *P *=* *0.0008; Fig. 5J) with available microarray data and GEP analysis. In this independent subset of DLBCL patients who were treated with either CHOP or R‐CHOP, the new model could stratify each group into cohorts with significantly different OS rates (Fig. 5H–J), which were similar with the training data stratified by the new algorithm or GEP method (Fig. 5B, C, E and F). In addition, our algorithm could divide 475 patients with R‐CHOP regimen into two groups with significantly different progression‐free survival (PFS) rates (HR, 0.596; 95% CI, 0.441 H–0.806; *P *=* *0.0007; Fig. 5L), which was also similar with GEP analysis (HR, 0.612; 95% CI, 0.445 H–0.842; *P *=* *0.0025; Fig. 5K). In brief, the DLBCL patients with GCB or Non‐GCB subtypes according to the eight‐marker model, did not differ significantly with GEP classification in terms of clinical characteristics at presentation in the validation cohorts.

### The classification model was independent of IPI as well as GEP analysis

**Figure 6:**
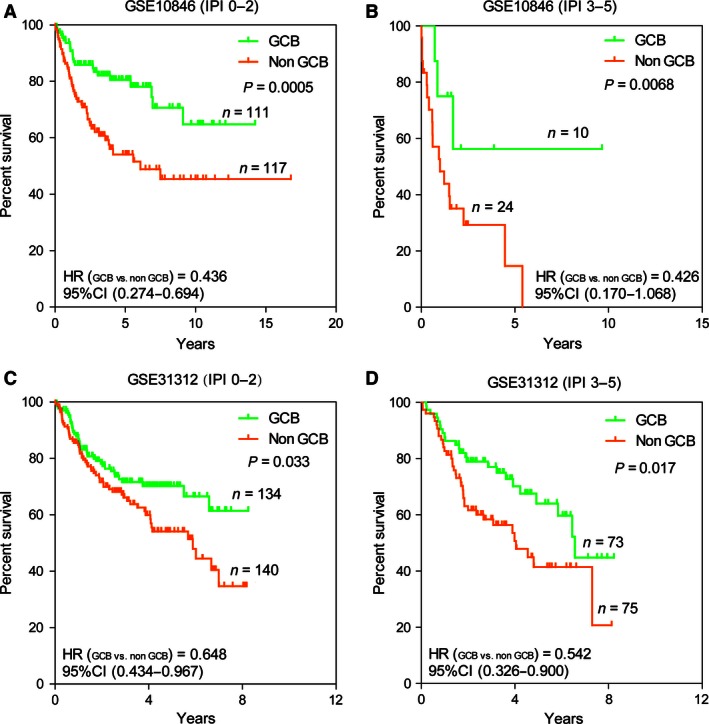
The eight‐gene model and the International Prognostic Index (IPI). The Kaplan–Meier estimates showed overall survival for groups of patients with low IPI scores and high IPI scores after stratified as GCB or Non‐GCB subtype on basis of the eight‐gene model. According to log‐likelihood estimates, *P *=* *0.0005 (A), 0.0068 (B) and *P *=* *0.033 (C), 0.017 (D) for the model based on a continuous variable applied to the GCB and Non‐GCB groups shown in the figure, respectively.

Subsequently, we studied whether the classification model could add a prognostic value beyond that of the IPI as well as GEP analysis. However, there were too few patients with high IPI scores (3–5) in GSE53786 for our results to achieve statistical significance. Therefore, we analyzed the larger data set published in GSE10846 to investigate the added value of the three‐gene model for IPI. Among clinical patients in our sample, we divided them into two groups according to their IPI score (low IPI: 0–2; high IPI: 3–5), and further subdivided the patients in each group into two subgroups (GCB and Non‐GCB subtypes) with significantly different OS rates (Fig. 6A–B) according to the classification from our new model. We also found that HR between GCB and Non‐GCB subgroup, as one of the important prognostic profiles, was around 0.5 in low (HR, 0.436; 95% CI, 0.274–0.694; *P *=* *0.0005) and high IPI group (HR, 0.426; 95% CI, 0.170–1.068; *P *=* *0.0068), which demonstrated that it would isolate the preferable or poor prognosis from the subgroups. When we combined the IPI score and the eight‐marker algorithm, we could identify a group of patients with a very favorable OS (IPI 0–2 and GCB phenotype, 5‐year OS rate of 80.5%) and a patients group with an unfavorable OS (IPI score 3–5 and Non‐GCB phenotype, 5‐year OS rate of 14.6%). Similarly, we validated the result in GSE31312 according to the subtypes predicted by the SVM model in low (HR, 0.648; 95% CI, 0.434–0.967; *P *=* *0.033) and high IPI group (HR, 0.542; 95% CI, 0.326–0.900; *P *=* *0.017; Fig. 6C–D). And we also obtained a cohort with good prognosis in IPI 0–2 subgroup (GCB phenotype, 5‐year OS rate of 70.2%) and a set of patients with poor prognosis in IPI 3–5 subgroup (Non‐GCB phenotype, 5‐year OS rate of 41.4%). Generally, we concluded that the new model could be used to predict survival of DLBCL patients independently and added the predictive power of the IPI.

### The classification model had strongly diagnostic power for each subtypes of DLBCL

**Figure 7:**
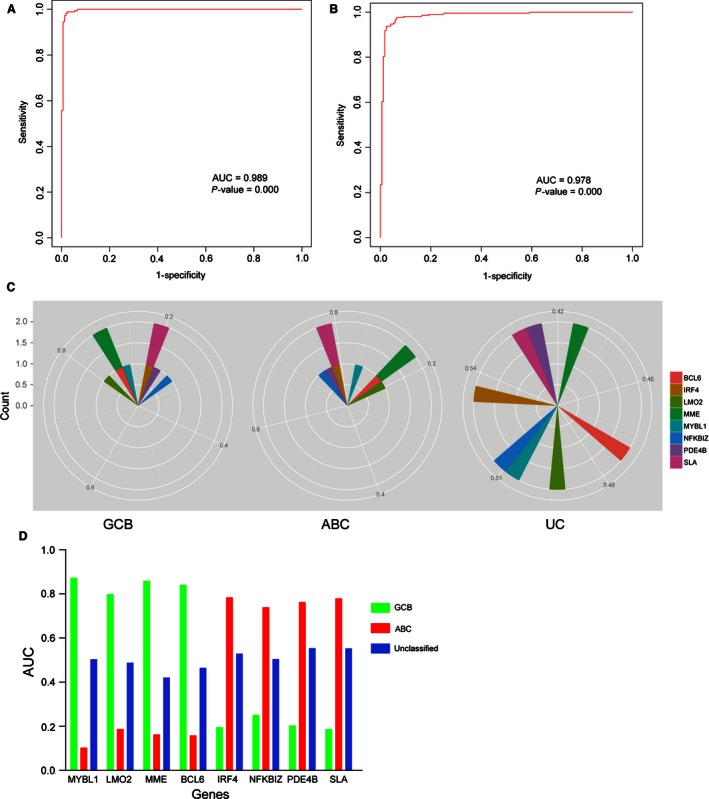
Access the discriminatory accuracy of the SVM model and each marker with ROC curve analysis. (A, B) The ROC curve analysis evaluated the discriminant power with large AUC area for training data (0.989, *P *=* *0.000) and validated data (0.978, *P *=* *0.000). (C) Radar map method was used for analyzing the distribution of the AUC values of each gene marker in assigning cases to GCB, ABC or unclassified (UC) classification. (D) The performance of each marker could be quantified by the area under the ROC curve. Based on these parameters, *MYBL1, LMO2, BCL6* and *MME* were the best marker for GCB subtype, and *IRF4, PDE4B, NFKBIZ* and *SLA* were more specific in recognizing ABC‐DLBCL.

To identify the diagnostic value in molecular subtypes, the ROC (receiver operating characteristic curve) method was used to compare the power between the training data and validation data. The area under the curve (AUC) of the new algorithm was 0.989 (*P *=* *0.000) and 0.978 (*P *=* *0.000), respectively (Fig. 7A–B). This indicated that the new algorithm could predict the molecular subtypes with high sensitivity and specificity, which demonstrated our new algorithm was sufficient to work as a practical clinical tool in the current therapeutic era.

From the ROC curve analysis, we could identify that the specificity and sensitivity of each marker in assigning cases to GCB or ABC classification. The performance of each marker could be quantified by the area under the ROC curve (AUC). As a result, we found that *MYBL1, LMO2, BCL6*, and *MME* genes were distributed in GCB subtype patients with higher AUC value (AUC >0.8, *P *=* *0.000); meanwhile the AUC value of the other four genes were more in ABC subtype patients (AUC >0.7, *P *=* *0.000; Fig. 7C–D). We concluded that the eight markers had high diagnostic power in the DLBCL molecular classification.

## Discussion

As a way utilized widely in clinical practice, some approaches based on IHC for “Cell of Origin” (COO) segmentation have been developed instead of GEP analysis by many clinical departments. However, recent studies have demonstrated that various iterations of the related algorithms are not associated well with each other 13 with low accordance of GEP analysis. So, a robust and reliable model for COO profiling applied into both research and clinical samples is required to discover and stratify the DLBCL subtypes precisely.

The most well COO subclassification methodology was established by Affymetrix microarray profiling 14. Our research confirmed the reliability of previous findings, indicating that GEP could be performed by extracting RNA from lymph node. In this study, we selected five GEO databases from the same platform and with the same data processing (MAS 5.0, Santa Clara, CA, USA) to construct the new model to validate the precise of the new algorithm. From the gene expression in the training data and validated data, a gene set (*MYBL1, LMO2, MME*, and *BCL6*) was significantly overexpressed in GCB subtype relative to the level of ABC; Inversely, the other set (*IRF4, PDE4B, NKFBIZ*, and *SLA*) was significantly more level in ABC than GCB group. The significantly different expression between GCB and ABC groups demonstrated the strong power for classification. Finally, a new algorithm based on the expression of the eight markers was designed to precisely stratify the GCB and ABC subtypes of DLBCL.

We evaluated the correlations of each gene with subtypes of DLBCL and the cutoffs to identify positively through ROC curves analysis. Compared with GEP analysis, the new model achieved 87% sensitivity, 90% specificity for ABC subtype and 92% sensitivity, 93% specificity for GCB subtype. OS rates were significantly different between the ABC and GCB subgroup classified by the new model. Patients with overexpression in the first gene set (*MYBL1, LMO2, MME*,* and BCL6*) could obtain more favorable prognosis than patients with the other set (*IRF4, PDE4B, NKFBIZ*,* and SLA*). There was a strong prognostic power for our new model to match with GEP analysis in CHOP/R‐CHOP treated patients. Also, our new model was independent of IPI as well as GEP analysis. We confirmed the new model's prognostic value in two independent cohorts from validated patients. At last, the new model gave us an opportunity to stratify the patients with unclassified subtype, although it does not reach statistical significance in the OS analysis.

To the best of our knowledge, *MYBL1* belongs to the Myb oncogene family of transcription factors, which are involved to regulate the proliferation and differentiation of distinct hemopoietic cells 15. So, *MYBL1* could be a specific marker for proliferating centroblasts because it is specifically induced in proliferation centroblasts 16. *MYBL1* located in the chromosome region 8q22 is involved in recurrent translocations in malignant lymphoma. We can infer that *MYBL1* could be a candidate for involvement in such locations 17.

Gene expression studies have reported that *LMO2* mRNA expression in DLBCL were part of the “germinal center” expression profile 3, and it is the strongest predictor of OS in DLBCL 18. Furthermore, *LMO2* expression has been associated with better overall survival in patients treated with CHOP/R‐CHOP 19. Unlike its role in leukemias, *LMO2* expression in DLBCL is not correlated with any somatic genetic alterations, but with the germline genetic variation 20.

*BCL6* is reported to be frequently translocated and hypermutated in DLBCL 21, 22, 23, and contributes to the pathogenesis of DLBCL. It encodes a sequence‐specific repressor of transcription, which interacts with several corepressor complexes to inhibit transcription. In GCB subtype cells, it negatively regulates the genes that function in differentiation, apoptosis, and cell cycle control, and up‐regulates the expression of some genes important for GC reactions through the expression of some miRNAs (e.g, miR155). The important function of BCL6 is to promote GCB cells proliferation rapidly in response to T‐cell‐dependent antigens 24 and tolerate the physiological DNA breaks required for immunoglobulin class switch recombination and somatic hypermutation without p53‐dependent apoptosis response. The new finding demonstrates that *BCL6* not only acts as a repressor, but is also capable of inducing expression of genes including the GC markers *LMO2 and MYBL1* 25.

MME, also known as the common acute lymphocytic leukemia antigen or neutral endopeptidase, is a cell surface zinc metalloendopeptidase 26. MME can cleave signal peptides at the cell surface, which affect cell proliferation, differentiation, and migration 27. MME can be used as a diagnostic marker for a variety of cancers, especially for DLBCL 28. In this study, MME is more expressed in the GCB‐ than ABC‐subtype. As such, reduced expression of MME is correlated with a less favorable outcome of DLBCL patients 27, 29, 30.

*IRF4,* is at the center of both the transcriptional program of B‐cell terminal differentiation and of ABC‐DLBCL. It is required during an immune response for lymphocyte activation. Interestingly, partly because of a positive feedback mechanism involving NF‐*k*B, CARD11, and IRF4 in B‐cell receptor‐dependent NF‐*k*B signaling, ABC subtype DLBCL cells are also addicted to IRF4 for survival 31. Physiologically, BCL6 expression is suppressed by IRF4 32, which further highlights the significance of IRF4 for facilitating survival in ABC subtype DLBCL (Ref). Recently, IRF4 has been proposed as a crucial regulator and potential therapeutic target in ABC subtype DLBCL 33.

PDE4B, as one of isoforms from the PDE4 (phosphodiesterases 4) family, was previously defined an expression signature of prognosis in DLBCL 34. Some research demonstrated that *PDE4B* was overexpressed in fatal or refractory tumors with poorer prognosis 34. PDE4B not only deactivates the second messenger cyclic adenosine 3′,5′monophosphate (cAMP), but abolishes its inhibitory effects in B lymphocytes. Hence, DLBCL patients with high PDE4B expression, contributing to their poorer prognosis, could be resistant to cAMP‐induced apoptosis associated with inhibition of the phosphatidylinositol 3‐kinase (PI3K)/AKT signaling pathway 34, 35. Our analysis emphasizes the important role of PDE4B in the diagnosis or treatment of DLBCL and suggests that clinically relevant PDE4B might be useful in DLBCL malignancies with increased expression.

*NFKBIZ* encodes I*k*B‐*ζ* (also known as MAIL), which regulates the nuclear factor‐*k* B (NF‐*k*B) pathway 36, 37, 38. Constitutive activation of NF‐*k*B pathway is a hallmark of the ABC subtype of DLBCL 39, 40, 41. A study reported that *IkB‐ζ* expression seemed to be controlled through NF‐*k*B signaling in the vast majority of ABC DLBCL cases, as its expression was induced by mutants identified in patient samples that activated the NF‐*k*B pathway 42. This finding was confirmed by gene set enrichment analyses, which showed that the IkB‐*ζ* target gene signature was enriched in a gene set that distinguishes ABC from other lymphoma subtypes, suggesting that these target genes are indeed expressed at higher levels in primary ABC DLBCL patient samples compared with other malignant lymphoma subtypes. Combined with our algorithm analysis, I*k*B‐*ζ* encoded by ABC DLBCL‐specific gene *NFKBIZ,* was essential for the expression of a specific set of NF‐*k*B target genes (CARD11, CD79A, CD79B, and MYD88), which were essential for ABC‐DLBCL patients' poor survival 43, 44, 45, 46.

*SLA* is one of the most interesting glucocorticoid (GC)‐regulated candidate genes, which encodes an adaptor protein that negatively regulates cellular signaling initiated by tyrosine kinases in several systems 47. In B cells, SLA reduces levels of the antigen–receptor complexes by adapting the E3 ubiquitin ligase c‐CBL to components of the complex and targeting them for degradation 48. Its well‐documented inhibitory role in lymphocyte signaling raised the attractive possibility that its induction might play a critical role in GC‐induced cell cycle arrest and/or apoptosis 47. Prednisone is one of components in the standard chemotherapy regimen for DLBCL patients. However, in the process of chemotherapy, the patients with ABC subtype have a poorer prognosis than GCB subtype, meanwhile the ABC‐DLBCL patients have a significantly higher level of SLA expression than GCB‐DLBCL patients. Therefore, we could infer that SLA may be the critical factor to induce the worse effect of chemotherapy.

In conclusion, we found that the expression of eight markers could be “less‐gene‐possible” combination to stratify DLBCL patients into GCB and Non‐GCB subtypes with high specificity and sensitivity. Also, our model could predict an outcome similar with that of GEP analysis in CHOP or R‐CHOP‐treated patients. The findings are used in the research and new clinical trial studies associated with DLBCL. We believe that the new algorithm will continually improve the performance of the former methods, and make a better classification of DLBCLs for further characterizing the pathways that identify each of the DLBCL subtypes and for exploring the efficacy of new drugs in different subtypes.

## Conflict of Interests

None declared.

## Supporting information


**Figure S1.** The prognostic power for the eight markers in 414 patients stratified into two groups by genes' cutoff values.

**Table S1.** Sources of Evidence for a panel of 57 genes whose expression predicts survival in Diffuse Large B‐Cell Lymphoma.
