# Supplementary figures and images for: Machine learning‐based classification of diffuse large B‐cell lymphoma patients by eight gene expression profiles
Source: Cancer Med. 2016 Feb 11;5(5):837–52. doi: 10.1002/cam4.650 (PMC4864813; doi:10.1002/cam4.650)

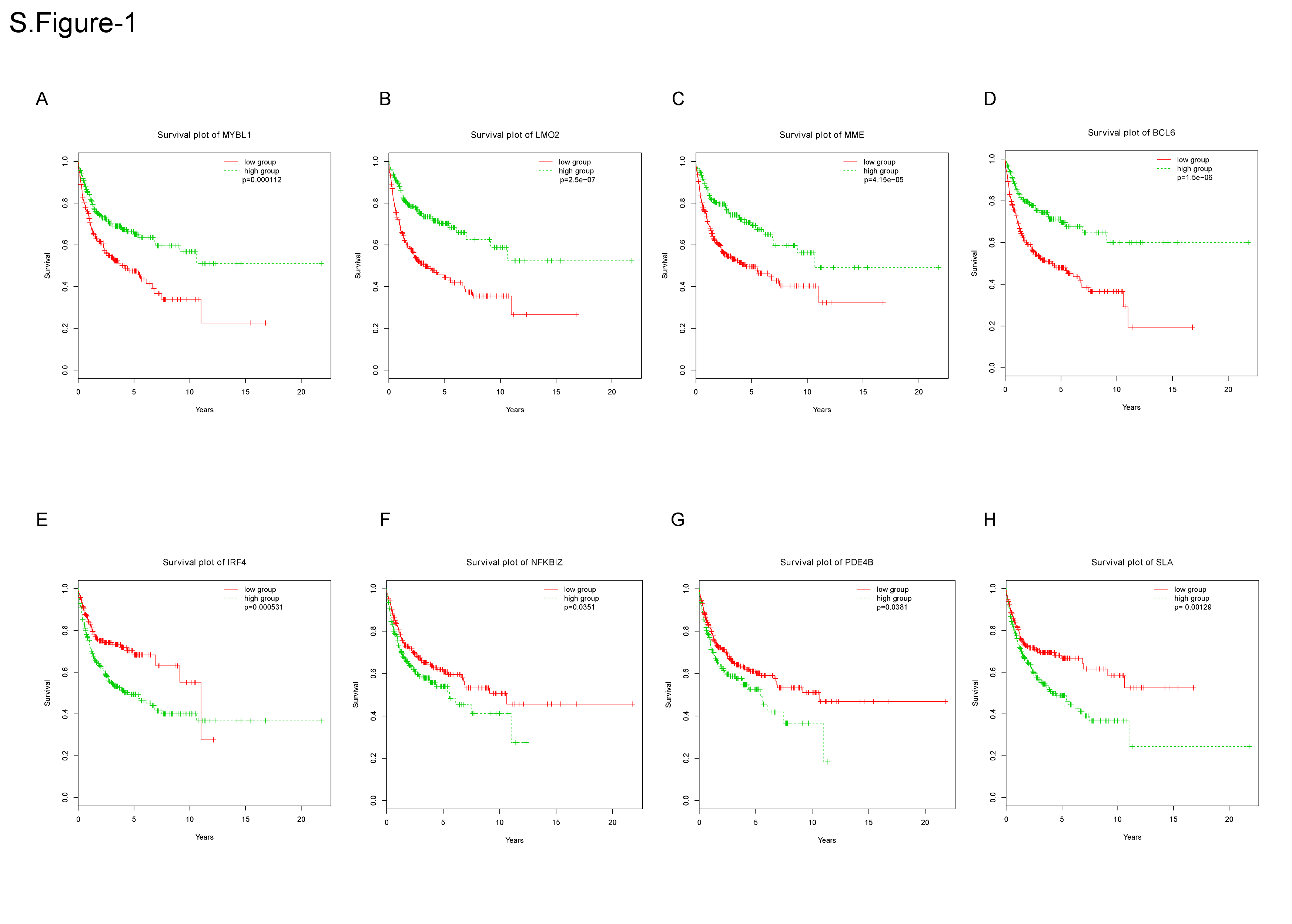

Supplement: Supplementary file 1 — Figure S1. The prognostic power for the eight markers in 414 patients stratified into two groups by genes' cutoff values. [file CAM4-5-837-s001.tif]

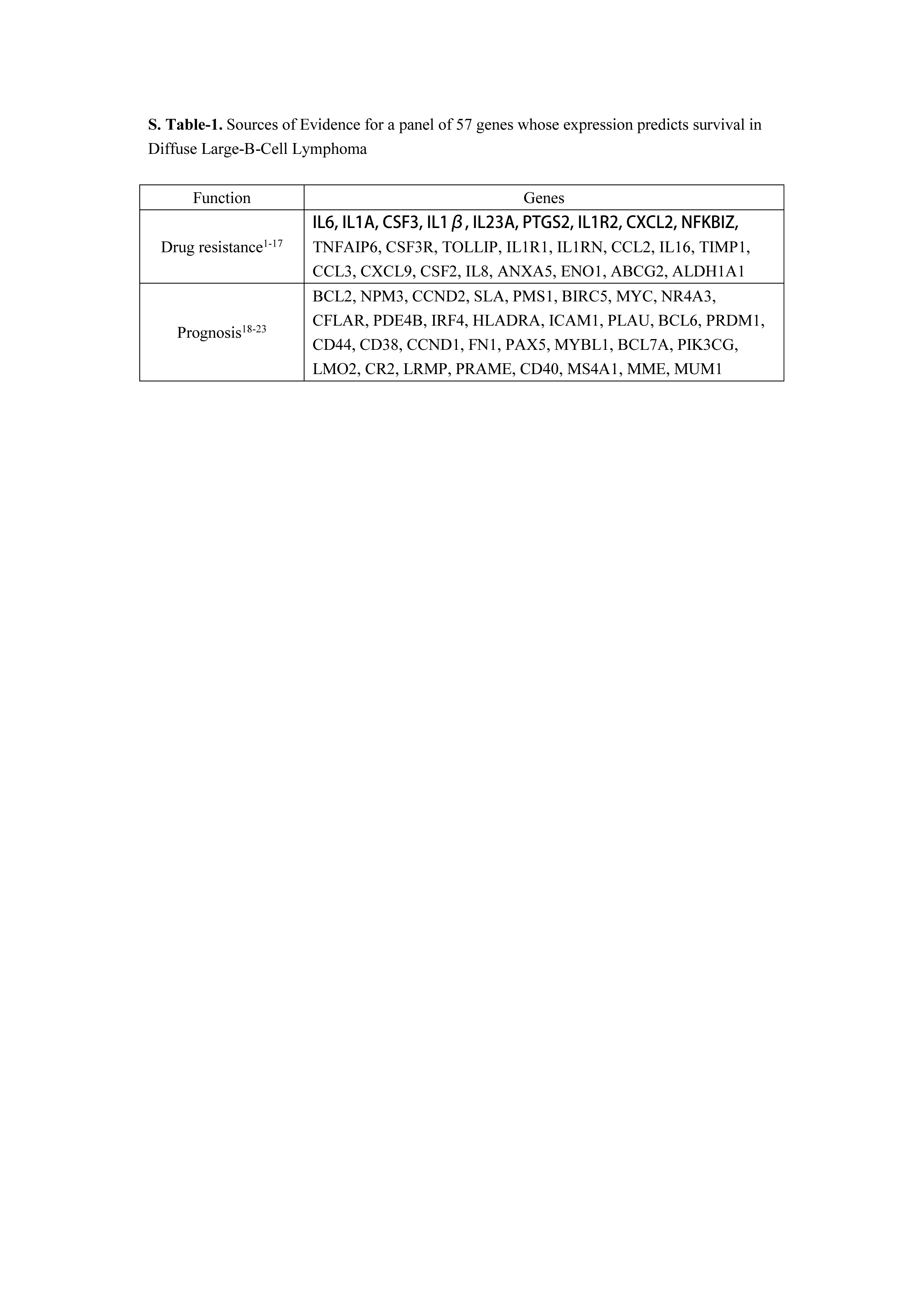

Supplement: Supplementary file 2 — Table S1. Sources of Evidence for a panel of 57 genes whose expression predicts survival in Diffuse Large B‐Cell Lymphoma. [file CAM4-5-837-s002.tif]
