# Supplementary material for: Machine learning‐based classification of diffuse large B‐cell lymphoma patients by eight gene expression profiles
Source: Cancer Med. 2016 Feb 11;5(5):837–52. doi: 10.1002/cam4.650 (PMC4864813; doi:10.1002/cam4.650)
Supplement: Supplementary file 3 [file CAM4-5-837-s003.docx]

| **S. Table-1.** Sources of Evidence for a panel of 57 genes whose expression predicts survival in Diffuse Large-B-Cell Lymphoma | |
| --- | --- |
| Function | Genes |
| Drug resistance[^1-17^](#_ENREF_1) | IL6, IL1A, CSF3, IL1β, IL23A, PTGS2, IL1R2, CXCL2, NFKBIZ, TNFAIP6, CSF3R, TOLLIP, IL1R1, IL1RN, CCL2, IL16, TIMP1, CCL3, CXCL9, CSF2, IL8, ANXA5, ENO1, ABCG2, ALDH1A1 |
| Prognosis[^18-23^](#_ENREF_18) | BCL2, NPM3, CCND2, SLA, PMS1, BIRC5, MYC, NR4A3, CFLAR, PDE4B, IRF4, HLADRA, ICAM1, PLAU, BCL6, PRDM1, CD44, CD38, CCND1, FN1, PAX5, MYBL1, BCL7A, PIK3CG, LMO2, CR2, LRMP, PRAME, CD40, MS4A1, MME, MUM1 |

**References**

1. Braganhol E, Kukulski F, Levesque SA, et al. Nucleotide receptors control IL-8/CXCL8 and MCP-1/CCL2 secretions as well as proliferation in human glioma cells. Biochim Biophys Acta 2015;1852:120-30.

2. Cao Y, Slaney CY, Bidwell BN, et al. BMP4 inhibits breast cancer metastasis by blocking myeloid-derived suppressor cell activity. Cancer Res 2014;74:5091-102.

3. Connolly EC, Saunier EF, Quigley D, et al. Outgrowth of drug-resistant carcinomas expressing markers of tumor aggression after long-term TbetaRI/II kinase inhibition with LY2109761. Cancer Res 2011;71:2339-49.

4. Garcia-Tunon I, Ricote M, Ruiz A, Fraile B, Paniagua R, Royuela M. IL-6, its receptors and its relationship with bcl-2 and bax proteins in infiltrating and in situ human breast carcinoma. Histopathology 2005;47:82-9.

5. Gay AN, Chang S, Rutland L, et al. Granulocyte colony stimulating factor alters the phenotype of neuroblastoma cells: implications for disease-free survival of high-risk patients. J Pediatr Surg 2008;43:837-42.

6. Korkaya H, Kim GI, Davis A, et al. Activation of an IL6 inflammatory loop mediates trastuzumab resistance in HER2+ breast cancer by expanding the cancer stem cell population. Mol Cell 2012;47:570-84.

7. Lee MJ, Heo SC, Shin SH, et al. Oncostatin M promotes mesenchymal stem cell-stimulated tumor growth through a paracrine mechanism involving periostin and TGFBI. Int J Biochem Cell Biol 2013;45:1869-77.

8. Liu D, Liu C, Wang X, Ingvarsson S, Chen H. MicroRNA-451 suppresses tumor cell growth by down-regulating IL6R gene expression. Cancer Epidemiol 2014;38:85-92.

9. Muerkoster S, Arlt A, Gehrz A, et al. Autocrine IL-1beta secretion leads to NF-kappabeta-mediated chemoresistance in pancreatic carcinoma cells in vivo]. Med Klin (Munich) 2004;99:185-90.

10. Ong PS, Chan SY, Ho PC. Microarray analysis revealed dysregulation of multiple genes associated with chemoresistance to As(2)O(3) and increased tumor aggressiveness in a newly established arsenic-resistant ovarian cancer cell line, OVCAR-3/AsR. Eur J Pharm Sci 2012;45:367-78.

11. St John MA. Inflammatory mediators drive metastasis and drug resistance in head and neck squamous cell carcinoma. Laryngoscope 2015;125 Suppl 3:S1-11.

12. Yang DR, Ding XF, Luo J, et al. Increased Chemosensitivity via Targeting Testicular Nuclear Receptor 4 (TR4)-Oct4-Interleukin 1 Receptor Antagonist (IL1Ra) Axis in Prostate Cancer CD133+ Stem/Progenitor Cells to Battle Prostate Cancer. Journal of Biological Chemistry 2013;288:16476-83.

13. Yang Y, Groshong JS, Matta H, Gopalakrishnan R, Yi H, Chaudhary PM. Constitutive NF-kappaB activation confers interleukin 6 (IL6) independence and resistance to dexamethasone and Janus kinase inhibitor INCB018424 in murine plasmacytoma cells. J Biol Chem 2011;286:27988-97.

14. Wang J, Zhang Y, Liu X, et al. Annexin A5 inhibits diffuse large B-cell lymphoma cell invasion and chemoresistance through phosphatidylinositol 3-kinase signaling. Oncology reports 2014;32:2557-63.

15. Zhu X, Miao X, Wu Y, et al. ENO1 promotes tumor proliferation and cell adhesion mediated drug resistance (CAM-DR) in Non-Hodgkin’s Lymphomas. Experimental Cell Research 2015.

16. Singh RR, Kunkalla K, Qu C, et al. ABCG2 is a direct transcriptional target of hedgehog signaling and involved in stroma-induced drug tolerance in diffuse large B-cell lymphoma. Oncogene 2011;30:4874-86.

17. Song Y-h, Zhong M-z, Gan P-p, et al. ALDH1A1 mediates resistance of diffuse large B cell lymphoma to the CHOP regimen. Tumor Biology 2014;35:11809-17.

18. Lossos IS, Czerwinski DK, Alizadeh AA, et al. Prediction of survival in diffuse large-B-cell lymphoma based on the expression of six genes. New England Journal of Medicine 2004;350:1828-37.

19. Mitsuhashi K, Masuda A, Wang Y-H, Shiseki M, Motoji T. Prognostic significance of PRAME expression based on immunohistochemistry for diffuse large B-cell lymphoma patients treated with R-CHOP therapy. International journal of hematology 2014;100:88-95.

20. Rydström K, Linderoth J, Nyman H, et al. CD40 is a potential marker of favorable prognosis in patients with diffuse large B-cell lymphoma treated with immunochemotherapy. Leukemia & lymphoma 2010;51:1643-8.

21. Suzuki Y, Yoshida T, Wang G, et al. Association of CD20 levels with clinicopathological parameters and its prognostic significance for patients with DLBCL. Annals of hematology 2012;91:997-1005.

22. Blenk S, Engelmann J, Weniger M, et al. Germinal center B cell-like (GCB) and activated B cell-like (ABC) type of diffuse large B cell lymphoma (DLBCL): analysis of molecular predictors, signatures, cell cycle state and patient survival. Cancer informatics 2007;3:399.

23. Li J, Hou J, Li L, Wang Y. Immunohistochemical subtypes of diffuse large B-cell lymphoma in the head and neck region. Genetics and molecular research: GMR 2015;14:3889.

**Figure legend**

**S. Figure-1. The prognostic power for the eight markers in 414 patients stratified into two groups by genes’ cutoff values.** The Kaplan-Meier analysis showed that patients with higher expression value of *MYBL1* **(A)**, *LMO2* **(B)**, *MME* **(C)** or *BCL6* **(D)** were significant favorable outcome. Oppositely, patients with higher expression value of *IRF4* **(E)**, *NFKBIZ* **(F)**, *PDE4B* **(G)** and *SLA* **(H)** were the undesirable result.
